# High-Tech Acupuncture and Integrative Laser Medicine

**DOI:** 10.1155/2012/363467

**Published:** 2012-09-04

**Authors:** Gerhard Litscher, Xin-Yan Gao, Lu Wang, Bing Zhu

**Affiliations:** ^1^Stronach Research Unit for Complementary and Integrative Laser Medicine, Research Unit of Biomedical Engineering in Anesthesia and Intensive Care Medicine, TCM Research Center Graz, Medical University of Graz, 8036 Graz, Austria; ^2^Institute of Acupuncture and Moxibustion, China Academy of Chinese Medical Sciences, Beijing 100700, China

Basic research on high-tech acupuncture and integrative laser medicine has been successfully performed all over the world during the last decades, using a broad spectrum of innovative biomedical engineering methods. One of the main goals is to combine basic research on high-tech acupuncture with necessary further experimental and clinical pilot studies for the first time. Acupuncture has been used for medical treatment for thousands of years. Using electroacupuncture, needle or laser needle stimulation, and modern biomedical techniques, it was possible for the first time to quantify changes in biological activities caused by acupuncture.

The patient is in China—the analysis for the efficacy of acupuncture is performed by experts in Europe. This *“transcontinental teleacupuncture”* is a way which was realized by our research team within joint Sino-Austrian and Sino-European projects. The investigations are carried out over thousands of kilometers; for example, 24-hour electrocardiographic (ECG) recordings from patients are registered in China, and the data are transferred directly after the acupuncture treatment to an analysis computer at the Medical University of Graz. The acupuncturists in China are informed about the results immediately based on the analysis protocol. 

These Sino-European studies included this special issue that contains 26 interesting publications, of which 12 are related to needle acupuncture, 5 to laser acupuncture, and 9 to electroacupuncture. Apart from body acupuncture, special emphasis is also given to auricular acupuncture. The investigations cover animal experimental studies and studies in healthy volunteers and patients, as well as basic and clinical research on evidence-based high-tech acupuncture, integrative laser, and translational medicine based on acupuncture and moxibustion. A first introduction of new developments of acupuncture stimulation equipment is also featured among the papers.

It has to be mentioned that this special issue has a total impact factor of 124.124 and contains, among others, the following topics: (i) modernization of acupuncture (evidence-based medicine, integrative laser medicine), (ii) high-tech acupuncture, (iii) development of innovative acupuncture stimulation methods (needle, laser, and electroacupuncture), (iv) methods for the quantification of peripheral and central effects of acupuncture, (v) scientific evaluation of complementary medical methods (acupuncture, acupressure, moxibustion, and laser therapy), (vi) computer-controlled acupuncture, (vii) teleacupuncture, (viii) laser needle acupuncture, (ix) red- and infrared laser stimulation, (x) violet laser acupuncture, (xi) biomedical assessment of acupuncture.

Modernization of acupuncture is a contemporary issue. The bridging between Eastern and Western medicine was successful using modern biomedical engineering technology, as described in this special issue. The next task is to make the arising possibilities and results useable for all involved persons.

